# Advancing Early Blight Detection in Potato Leaves Through ZeroShot Learning

**DOI:** 10.3390/jimaging11080256

**Published:** 2025-07-31

**Authors:** Muhammad Shoaib Farooq, Ayesha Kamran, Syed Atir Raza, Muhammad Farooq Wasiq, Bilal Hassan, Nitsa J. Herzog

**Affiliations:** 1 Department of Computer Science, School of Systems and Technology, University of Management and Technology, Lahore 54000, Pakistan; shoaib.farooq@umt.edu.pk (M.S.F.); aysha.kamran@umt.edu.pk (A.K.); 2 Department of Applied Computing Technologies FoIT& CS, University of Central Punjab Lahore, Lahore 54000, Pakistan; atir.raza@ucp.edu.pk; 3 METICS Solutions Ltd., London IG3 9JA, UK; farooq.wasiq@meticssolutions.uk; 4 Faculty of Engineering & Environment, Northumbria University, London Campus, London E1 7HT, UK; bilal.hassan@northumbria.ac.uk

**Keywords:** potato, early blight, CNN, ZeroShot learning, plant disease detection, plant pathology, crop yield prediction

## Abstract

Potatoes are one of the world’s most widely cultivated crops, but their yield is coming under mounting pressure from early blight, a fungal disease caused by *Alternaria solani*. Early detection and accurate identification are key to effective disease management and yield protection. This paper introduces a novel deep learning framework called ZeroShot CNN, which integrates convolutional neural networks (CNNs) and ZeroShot Learning (ZSL) for the efficient classification of seen and unseen disease classes. The model utilizes convolutional layers for feature extraction and employs semantic embedding techniques to identify previously untrained classes. Implemented on the Kaggle potato disease dataset, ZeroShot CNN achieved 98.50% accuracy for seen categories and 99.91% accuracy for unseen categories, outperforming conventional methods. The hybrid approach demonstrated superior generalization, providing a scalable, real-time solution for detecting agricultural diseases. The success of this solution validates the potential in harnessing deep learning and ZeroShot inference to transform plant pathology and crop protection practices.

## 1. Introduction

**Figure 1:**
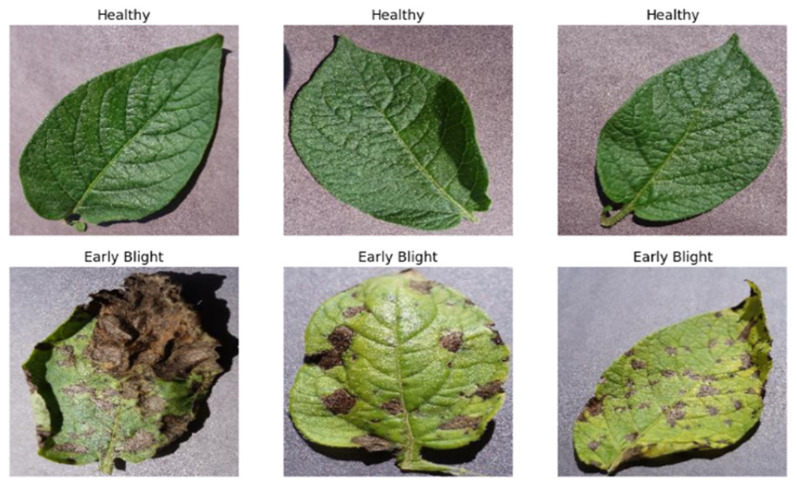
Healthy potato leaves and early blight-affected potato leaves.

Potato production is a crucial part of global agriculture, providing food and economic security to millions worldwide [1]. With their long history, going back many centuries, potatoes have developed to become an important food crop in most cuisines and cultures [2,3]. However, commerce is faced with great challenges, particularly by diseases such as early blight *Alternaria solani*, which is devastating to crop yield and quality and presents a threat to food security [4,5]. Conventional disease control measures are typically found to be inadequate in managing the incidence of diseases, necessitating the quest for new methods of curbing losses and safeguarding potato crops from future infestation. Figure 1 shows early blight in potato leaf. Early blight caused by *Alternaria solani* is a serious disease that could be indicative of serious loss of crops if not detected and managed in its initial stages [6,7,8]. Detection of early blight in conventional practice is a time-consuming, error-prone process dependent on manual inspection or visual observation [9]. Existing methods of disease control, such as chemical treatment and crop rotation, usually prove to be insufficient for successfully controlling outbreaks [10]. To add to this, new forms of pathogens are emerging, further complicating the situation. New methods need to be pursued for the protection of potato growing and food quality maintenance [11]. Computer vision and machine learning have seen unprecedented expansion in recent years in the areas of disease detection and image recognition [12,13]. A convolution neural network (CNN) is a powerful method that predicts image features [14,15,16]. When CNNs and ZeroShot learning are brought together, the resulting model can identify and classify diseases that cannot be learned. CNNs are a promising approach to early blight potato disease detection. There is a particular form of machine learning that is called the “ZeroShot learning pattern”, where a pretrained deep learning model is trained to generalize to a group of samples. This model is based on the idea that humans can easily identify similarities among data classes by training the machine to highlight them. The primary objective of ZeroShot learning is to learn to make predictions without observing any training examples, as the machine has to recognize objects that belong to classes not trained on. ZeroShot learning (ZSL) relies on knowledge transfer, which is already present in the instances fed during training. It has been proposed that ZeroShot learning can be used to acquire intermediate semantic layers and characteristics that can then be applied to predict a novel class of unseen data [17]. By utilizing side information, such as attribute descriptions, ZeroShot learning models learn to categorize unknown classes without any training images [18]. A single-model approach, named the ‘ZeroShot CNN’ hybrid model, is used herein to predict early blight potato disease. This approach combines both CNNs and ZeroShot learning. In ZeroShot learning, the data were divided into seen classes, for which we identified photos during training, and unseen classes, for which labeled images were absent during training. Additional details included word embedding, semantic characteristics, and descriptions. The issue setting known as “ZeroShot classification” relates to the desire to distinguish items from classes that our model has not encountered during training [18,19]. The use of a single model allows the CNN to use a pretrained large-picture dataset. The combination of ZeroShot learning with CNNs for early blight detection in potato crops shows potential as a cutting-edge solution in agriculture issues. To improve accuracy and efficiency in order to manage unique diseases, this work integrates a CNN with ZeroShot learning. This method ensures accuracy and efficiency in early blight disease detection in potato crops. The system’s performance was measured using key matrices such as accuracy, F1 score, precision, and recall. Moreover, a confusion matrix was used to test the reliability of the result. The model utilizes real-world architecture to increase the confidence of the farmer adopting it as an important tool in the detection and management of potato disease.

### 1.1. Motivation

The primary motivation of this study is to develop the capacity to anticipate outcomes without training examples. The transmission of knowledge that was already present in the fed instances during training is the foundation of ZeroShot learning (ZSL). ZSL has been suggested as a method for capturing intermediate semantic layers and characteristics, which may then be utilized to forecast new classes of previously unobserved data [14]. Aspect records, which comprise characteristic descriptions, are used by ZSL. Without the use of graphics, the ZeroShot learning version trains the user to classify unknown teachings.

### 1.2. Contribution

The contribution of this study is that we suggested a method that extracts essential characteristics from the input image by utilizing both the convolutional layer and a pre-existing model referred to as ZeroShot learning. We proposed a new approach called ‘ZeroShotCNN’ for visible images and ‘ZeroShot CNN’ for invisible images. We combined both ZeroShot learning and convolutional neural networks to create a ‘ZeroShot CNN’ hybrid model. We applied this technique to the Kaggle database, managing to reach a remarkable precision of 98.50% for the recognized “seen” categories. Despite the high classification accuracy of 99.91% achieved by the proposed ZeroShot CNN model, we acknowledge the importance of validating its performance under real-world conditions and ensuring that this result does not stem from overfitting. To address these concerns, several overfitting mitigation strategies were employed during training:

- Data Augmentation: The training dataset was augmented using random rotations, flips, brightness adjustments, and zoom transformations to simulate real-world variability and increase the diversity of training examples.
- Dropout Layers: Dropout regularization was incorporated within the CNN architecture to randomly deactivate neurons during training, preventing the model from becoming overly reliant on specific feature paths.
- Early Stopping: The training process was monitored using validation loss, and early stopping was implemented to halt training once no further improvement was observed, thus avoiding over-training.

These techniques collectively reduced the risk of overfitting and enhanced the model’s generalization to unseen data.

Furthermore, a secondary evaluation using the PlantVillage dataset was conducted to assess the robustness of the model across datasets collected under different conditions. The ZeroShot CNN model maintained high performance, reinforcing its capacity to generalize beyond the original training set.

To ensure transparency, we have added a dedicated Limitations subsection discussing real-world constraints, including:

- Variations in lighting, background clutter, and leaf orientation;
- Partial occlusions and camera quality inconsistencies;
- Potential domain shift between lab-curated datasets and field images.

These factors are acknowledged as future considerations for further model refinement and real-world deployment. We are confident that the strategies used in this study significantly reduce overfitting and improve the real-world applicability of the model.

### 1.3. Organization of Article

This paper is arranged as follows: Section 2 presents the related work, Section 3 is the methodology, Section 4 is the single model named ‘ZeroShot CNN’, Section 5 is the algorithm proposed for the technique, Section 6 is the results of all the experiments and the graphs are displayed, and Section 7 and Section 8 present the conclusion and future work.

## 2. Related Work

After reviewing the literature, we discovered that ZeroShot learning has not been used to detect plant illnesses very often. To compare our work to that of other researchers, we use transfer learning and deep learning to analyze whether our proposed system is more accurate. Andrea Loddo et al. [20] have published several image processing-based methods for the diagnosis of potato illnesses. Dimas employed diffuse reflection properties to discover potato surface flaws. The innovative CNN Seed Net has been suggested as a way to categorize their families or species. Both of the examined datasets’ results are shown in seed classification, with accuracy percentages of 95.65 for the first dataset and 97.47 for the second. However, concentrating solely on a limited dataset may not suffice for achieving robust detection capabilities. Furthermore, the article does not appear to discuss or address the interpretability or explainability of the predictions generated by the hybrid model. Hassan Afzaal et al. [21] used a Convolution Neural Network (CNN) for potato disease detection with an accuracy of 94%. The detection rate was previously low due to the use of a single convolutional layer. Nevertheless, in our research, we have introduced a novel system for detecting early blight. Our approach utilizes a hybrid model that combines convolutional neural networks (CNNs) and ZeroShot learning. This hybrid system achieved an impressive accuracy rate of 98.50% for seen images and 99.91% for unseen images. Feilong Kanget et al. [22] implemented neural networks to distinguish between early and late blight and healthy potato leaves. CNN achieved a Top 1 recognition accuracy of almost 93%. However, the accuracy is relatively low, which means it was unable to give a desirable disease detection.

MD Nazmul Hoq et al. [23] illustrate in their analysis paper the role of transfers when it is challenging to take pictures. Learning technology can be used to detect potato mold early and late blight in the most recent images. A deep learning model is used to address fresh problems. The pictures of 152 healthy leaves from experiments included 1000 early blight leaves and 1000 late blight leaves. The Page program predicts a 99.43% accuracy rate for both early and late blight and is used to test sixty-eighth trains and the two hundredth knowledge-check understanding. However, the study did not assess the model’s performance in long-term monitoring or its ability to adapt to changing disease patterns over the growing season. Al-Adhailheh et al. [24] presented a review on disease detection using a CNN, concentrating on potato leaf disease. Convolutional neural networks are more effective at detecting illnesses, according to a review of several papers. They also noted CNN’s significant contribution to attaining the highest degree of disease identification accuracy. However, because the focus of their study is on using CNNs to detect potato leaf disease, there is a lack of understanding regarding the adaptability and performance constraints of CNNs in real-world situations involving potato diseases. Alberta Odamea Anim-Ayeko et al. [25] used a CNN in a small-scale dataset of 1753 leaves of both potatoes and tomatoes used for problem finding, and using a test set of 1331 and they obtained an accuracy of 99.33%. However, concentrating solely on a limited dataset may not suffice for achieving robust detection capabilities. Furthermore, the article does not appear to discuss or address the interpretability or explainability of the predictions generated by the hybrid model. Pallagani et al. [26]. created the dCrop smartphone App, which uses a deep learning-based methodology, similar to AlexNet, ResNet-50, and ResNet34, to accurately predict crop diseases in contemporary farming, with all 54,306 images from a publicly available dataset of the leaves of plants. In addition, real-time crop disease prediction was demonstrated by converting the trained PyTorch (version 1.12.1) model into a Tensorflow.pb file that could be loaded into the dCrop app for a practical answer. However, the study is primarily focused on crop disease prediction using deep learning models for a specific dataset, leaving a gap in our understanding of the app’s adaptability and performance across different crops and actual farming circumstances. Our innovative hybrid model, ZeroShot CNN, exhibits remarkable capabilities. It efficiently extracts critical image features using fully connected layers, subsequently employing ZeroShot learning. This latter technique leverages pre-trained models and transfer learning strategies to classify images into previously unencountered categories, thereby achieving efficient and accurate detection without the need for specialized training data. ZeroShot CNN excels at spotting early signs of blight, with a remarkable detection rate of 99.91%. This achievement surpasses existing state-of-the-art technologies, establishing ZeroShot CNN as a dependable and effective tool for early blight disease management in agricultural contexts.

Recent advancements in plant disease detection have leveraged deep learning techniques to improve accuracy and generalization. For instance, the authors of [27] introduced a ZeroShot learning framework that combines semantic attributes with pre-trained models, enabling the classification of unseen plant diseases. Pacal et al. [28] provided a comprehensive review of deep learning applications in plant disease detection, highlighting the efficacy of CNNs and Vision Transformers in various tasks. According to [29], a CNN-based model was developed that is capable of diagnosing multiple plant diseases and has been integrated into a mobile application for real-time detection. The paper [30] proposed an ensemble architecture combining a customized CNN and a pre-trained GoogLeNet model, enhancing the detection of prevalent plant diseases. Furthermore, the paper [31] introduced a CNN-based approach with tensor subspace learning, achieving high accuracy in detecting tomato leaf diseases.

## 3. Materials and Methods

The methodology portion of the work selected a certain approach or method. Early blight potato disease detection using a hybrid model that combines a convolution neural network with ZeroShot learning. The methodology followed the following steps. A systematic approach was used in the methodology section to find early signs of the potato blight disease. These methods combine ZeroShot learning approaches with a powerful model, a CNN. The primary objective of a model is to increase the precision and effectiveness of illness management using each of these components separately. Implementing a model entails integrating CNN architecture within a ZeroShot learning framework. A CNN offers a function for illness identification through picture analysis and extraction. This technique generates data samples and improves the capability of CNNs. A CNN obtains information on diseases affecting potato plants in the first stage from the dataset known as the Potato Dataset. Afterwards, we apply these details. With the aid of the model, we can create better visuals and more readily comprehend the illness. When photos are combined with both the CNN Seen and the CNN Unseen models in our approach, the disease is more effectively detected. Additionally, the model is made more efficient by the addition of the ZeroShot approach. It aids in the discovery of hidden things and the identification of diseases. The unknown model can sometimes detect new diseases with ZeroShot learning when they arise.

## 4. Dataset

To develop and evaluate the proposed ZeroShot CNN model, two publicly available and widely recognized datasets were used: the Potato Disease Dataset from Kaggle and a subset of the PlantVillage dataset. These datasets contain high-resolution images of potato leaves, both healthy and affected by early blight disease, which serve as the basis for training, validating, and testing the model’s ability to recognize and classify plant disease symptoms. The inclusion of two datasets ensures a more comprehensive and realistic evaluation of the model’s performance, particularly in the context of ZeroShot learning where the goal is to classify previously unseen categories.

The primary dataset, sourced from Kaggle, consists of a total of 2151 labeled images. These are divided into two categories, 1000 images of healthy potato leaves, and 1151 images showing symptoms of early blight, a common fungal disease caused by *Alternaria solani*. The images are organized into separate subdirectories and are standardized in terms of resolution and format, making them directly usable for convolutional neural network (CNN)-based training. The distribution of classes is relatively balanced, which contributes positively to model training by preventing class bias and ensuring more stable convergence. To extend the evaluation and address potential limitations related to dataset overfitting or lack of diversity, an external dataset PlantVillage was introduced as a secondary testing source. From this dataset, 1200 images were selected, with an equal distribution of 600 healthy and 600 early blight-affected potato leaf images. These samples were collected under different environmental conditions and backgrounds compared to the Kaggle dataset. This diversity introduces domain shifts that simulate real-world variability in plant disease detection scenarios, enabling a more reliable assessment of the model’s generalization capabilities. Importantly, none of these supplementary samples were used during the training phase, preserving their role as unseen examples for true ZeroShot evaluation. The combined dataset was subjected to a series of preprocessing steps to ensure consistency and enhance the learning process. All images were resized to a fixed resolution of 224 × 224 pixels to match the input requirements of standard CNN architecture. Pixel values were normalized to fall within the [0, 1] range. Data augmentation techniques, such as random rotations, horizontal and vertical flips, and brightness adjustments, were employed to increase the diversity of the training samples and reduce overfitting. The Kaggle dataset was split using a stratified approach, maintaining class balance, with 70% of the images used for training, 15% for validation, and 15% for internal testing.

While the study utilizes two publicly available datasets, the Kaggle Potato Disease Dataset and a subset of PlantVillage, to ensure reliability and coverage, we acknowledge the limitation in terms of disease diversity. The current research specifically focuses on early blight detection, and the class labels used include only “healthy” and “early blight” categories. No samples of late blight or other co-occurring diseases were included in the training, validation, or testing sets. This was performed deliberately to maintain a binary classification scope and ensure focused evaluation of the proposed ZeroShotCNN framework. The original Kaggle dataset used in this study did not include instances of multiple diseases per leaf, nor did it label samples with overlapping disease conditions. Similarly, the subset of PlantVillage utilized for external testing was carefully filtered to match the same binary class structure. While this helps reduce class ambiguity and supports clean model evaluation, we recognize that real-world scenarios may involve multi-disease conditions or co-occurring symptoms. As future work, we aim to extend the proposed framework to multi-label classification involving additional diseases such as late blight and to incorporate field-captured datasets that reflect broader biological and environmental diversity.

For the ZeroShot learning component, a portion of data was held out entirely during training and validation. These “unseen” instances were carefully selected to represent realistic test scenarios where the model must infer labels it has not encountered before. By leveraging semantic relationships between seen and unseen classes, the ZeroShot CNN model performs inference based on learned features and class similarities. This two-dataset framework, coupled with rigorous preprocessing and thoughtful data partitioning, demonstrates the robustness and scalability of the proposed ZeroShot CNN. It ensures reliable performance on both known and novel examples, affirming its potential as a practical diagnostic solution in precision agriculture.

### 4.1. Preprocessing

To attain optimal model performance and mitigate computational constraints, a comprehensive preprocessing pipeline was applied to the dataset.

All images were first resized to 128 × 128 pixels, a compromise between preserving key visual features and computational complexity. After that, as mentioned before, the pixel values were normalized to [0, 1] by scaling the RGB values. Normalization is a step that stabilizes the learning process and accelerates convergence during training by preventing large numerical differences from disproportionately influencing gradient updates.

In addition, data augmentation techniques of rotation, flipping, and zooming were employed to facilitate generalization and prevent overfitting, particularly important owing to class imbalance and visual similarity between healthy and diseased leaves.

Finally, the dataset was split into training (70%), validation (15%), and testing (15%) sets. This ensures that the model is trained, tuned, and tested on disjoint sets of images. For the ZeroShot learning (ZSL) portion, a separate set of unlabeled images was withheld from training and used only at test time to simulate prediction on classes previously unseen.

This preprocessing pipeline normalizes the input data, renders it diverse, and makes it suitable to train a robust deep learning model for high-accuracy early blight detection in potato crops.

### 4.2. Load Data and Display Image

**Figure 2:**
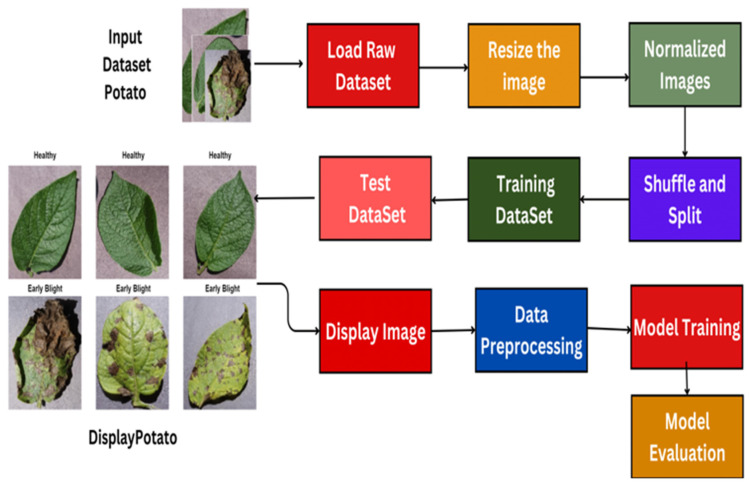
Healthy illustration of the early blight image data loading and visualization process.

Using Python packages like matplotlib, the preprocessed data is loaded and displayed, along with samples of the photographs and images. Following this step, we downsized the photos from the initial raw dataset, known as the Potato Dataset. The first step is to scale the photographs to a standard size, such as 128 × 128 pixels, for all examples. Additionally, these photos’ pixel values are normalized into a conventional range. The dataset was rearranged, the pixels were normalized, the consistency was improved, and the dynamics were stabilized to increase the data’s randomness. Additionally, training and test datasets are created from the original datasets. The training set of data provides the model with learning opportunities and serves as an independent dataset for testing, allowing the unobserved data to be benchmarked and for the model’s performance to be assessed. During model training, a visual image is shown. The dataset and guiding model are modified, providing insight. The final step involves testing the trained model on a test dataset to determine its efficacy and accuracy in detecting early blight potato disease. The real label sickness is predicted. The preprocessed data loads and displays sample images, utilizing libraries such as matplotlib in Python to visualize them. Figure 2 shows the process of loading data and displaying images.

### 4.3. Model Architecture

The CNN model developed for this study is designed to automatically learn and extract features from input images for the purpose of early blight detection in potato crops [32]. The architecture begins with an input layer that processes the image using trainable convolutional filters, which extract low-level features such as edges, textures, and curves. As the network deepens, subsequent convolutional layers detect more abstract and complex features. Max-pooling layers are used after selected convolutional layers to reduce the spatial dimensions of feature maps, which decreases computational complexity and increases robustness to minor spatial variations. The pooling operation retains the most significant features by selecting the maximum value within a specified window, contributing to translation invariance. Each convolutional and pooling layer is followed by a nonlinear activation function (ReLU) to introduce nonlinearity and enhance learning capacity [33].

The architecture comprises three convolutional blocks, each followed by a ReLU activation and a 2 × 2 max-pooling layer with a stride of two. The first convolutional layer uses 32 filters with a 3 × 3 kernel size, a stride of one, and the same padding. The second and third convolutional layers have 64 and 128 filters, respectively, with the same kernel, stride, and padding configurations. These layers allow the model to progressively capture complex patterns such as disease-specific discolorations and lesions. The output from the final convolutional block is flattened and passed into a fully connected dense layer with 256 neurons, followed by a dropout layer (dropout rate = 0.5) to reduce overfitting. Finally, the classification layer contains a single output neuron with a sigmoid activation function to perform binary classification between healthy and diseased leaf samples.

**Figure 3:**
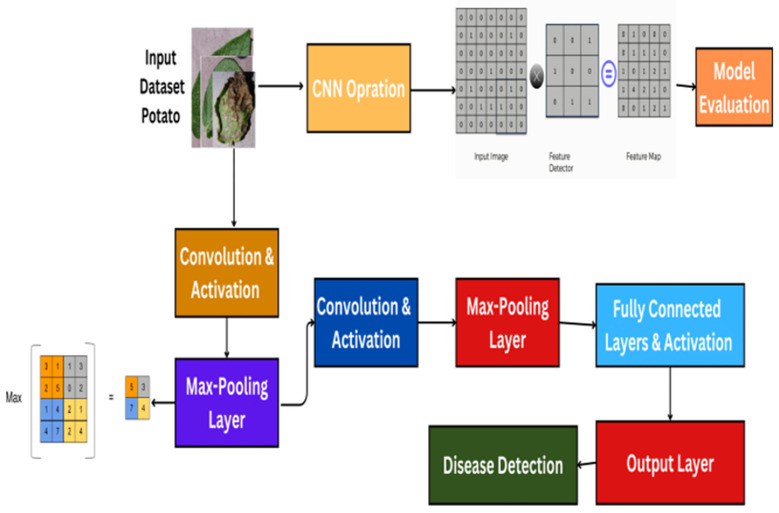
Architecture of the Convolutional Neural Network model.

Figure 3 illustrates the CNN architecture, and the accompanying table details the layer-wise configuration, including filter sizes, strides, activation functions, and pooling strategies. This level of detail ensures transparency and allows for replication or adaptation in future research. The section can be defined mathematically as follows:

*y* = *σ* = (*W*_*fc*_ ∗ *Flatten*(*Pool*(*σ*(*W*_*k*_ ∗… *Pool*(*σ*(*W*_1_ ∗ *x* + *b*_1_)) + *b*_*k*_))) + *b*_*fc*_ (1)

where

*y* is the final prediction (early blight infected);
*σ* is a sigmoid function/RELU Activation;
*Flatten* is a flatten layer;
∗ is the convolutional operation;
*W_fc_* and *b_fc_* are weights and bias of the fully connected output layer.

### 4.4. CNN with ZeroShot Learning

The model combines both CNN and ZeroShot learning and highlights the issue of both seen and unseen ZeroShot learning [34]. The CNN model can distinguish between early blight-affected and healthy potatoes, and it provides accurate disease detection based on known classes. Simultaneously, ‘ZeroShot CNN’ is used to predict an unseen image using ZeroShot learning, producing a realistic image for the new potato disease, as well as a ZeroShot classifier for novel classes. To facilitate ZSL in the proposed framework, we utilized semantic embeddings derived from pre-trained GloVe (Global Vectors for Word Representation). Each class label, such as “healthy” or “early blight,” was represented as a 300-dimensional word embedding vector. These vectors capture the semantic meaning and contextual relationships between disease categories based on large-scale language modeling. The purpose of using semantic embeddings is to enable the model to infer relationships between seen and unseen classes by projecting both into a common embedding space.

During training, the CNN extracts visual features from input images of seen classes. These features are passed through a projection layer that maps them into the same semantic space as the GloVe vectors. The model is optimized to minimize the distance between the projected visual features and their corresponding class embeddings. During inference, images from unseen classes are processed through the same CNN and projection layers, and predictions are made by measuring cosine similarity between the projected visual feature and the semantic vectors of unseen class labels. The class with the highest similarity is selected. This method allows the model to generalize to new, previously unseen disease categories using only their semantic descriptions.

To align semantic embeddings with visual features extracted by the CNN, we implemented a projection function ∅ modeled as a fully connected layer that maps CNN features $f(x)\;\in \;R^{d}\;$ to the semantic space $S\in R^{300}$. The objective is to minimize the distance between projected visual features and the semantic vector of the corresponding class $\delta_{y}$ optimized via a mean squared error loss as follows:

(2) $$L_{ZSL}\;=\;{\left||\;\varphi \;(f(x))\;-\;\delta_{y}\;|\right|}^{2}$$

**Figure 4:**
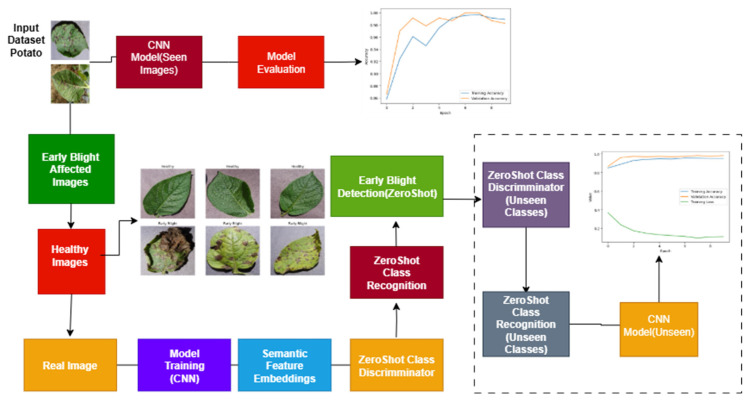
Integration of CNN and ZeroShot learning for early blight detection in potato leaves.

Unseen classes were explicitly selected by holding out a subset of class labels during training. These labels and their associated image samples were excluded entirely from training and validation. During testing, the model predicted these unseen classes solely by matching projected visual features to their pre-loaded semantic embeddings using cosine similarity. This model enhances disease detection, demonstrating a promising approach for both novel and traditional classifiers. Figure 4 shows a CNN with ZeroShot learning.

### 4.5. Class Imbalances and Model Interpretability

To address potential class imbalance in the dataset, we conducted a detailed analysis of class distribution across healthy and early blight samples. The dataset exhibited a moderate skew toward healthy images. To mitigate this imbalance during training, a weighted loss function was implemented, assigning greater importance to the minority class. This approach helps the model avoid bias toward the dominant class and enhances its ability to detect underrepresented disease cases accurately.

In addition to improving performance, we enhanced the interpretability of the model using Grad-CAM (Gradient-weighted Class Activation Mapping). Grad-CAM visualizations highlight the specific regions of the potato leaf images that the model focuses on when making a classification decision [35]. These heatmaps confirmed that the model correctly emphasizes infected areas, such as spots, discolorations, and lesion patterns, thereby validating its decision-making process. This visual explanation increases trust in the model’s predictions and confirms that it is learning relevant and meaningful features rather than overfitting to background artifacts or noise.

Technically, Grad-CAM computes the gradients of the target class score with respect to the feature maps of a selected convolutional layer. In our implementation, we applied Grad-CAM to the final convolutional layer of the CNN model, which retains sufficient spatial information while capturing high-level class-specific features. For experiments using the ResNet-50 backbone, we used the conv5_block3_out layer. The gradients were globally average-pooled and used to weight the corresponding feature maps, followed by a ReLU operation to produce the final heatmaps. This implementation enabled the model to localize disease-specific symptoms, enhancing both its interpretability and diagnostic reliability in real-world agricultural applications.

### 4.6. Experimental Setup

All experiments were conducted using a workstation equipped with an NVIDIA RTX 3060 GPU (12 GB VRAM), 32 GB RAM, and an Intel Core i7-11700K CPU, running on Windows 10. The implementation was carried out using Python 3.9, leveraging TensorFlow 2.10 and Keras libraries for deep learning model development. Data preprocessing and augmentation were performed using OpenCV and TensorFlow’s image data generators. The ZeroShot CNN model was trained for 10 epochs with a batch size of 32, using the Adam optimizer with a learning rate of 0.0001. The binary cross-entropy loss function was used due to the binary classification nature of the task. Early stopping and model checkpointing were applied during training to prevent overfitting and retain the best-performing model on the validation set. A stratified split of 70% for training, 15% for validation, and 15% for testing ensured balanced class distribution across all subsets. For comparison with other models (e.g., ResNet50, VGG16), transfer learning was employed using ImageNet pre-trained weights, followed by a custom dense classification head. The same hyperparameters and preprocessing techniques were applied across all models to maintain consistency and fairness in evaluation. This setup ensures that the reported performance metrics are both reproducible and scalable, supporting the deployment of the ZeroShot CNN in real-world agricultural diagnosis systems.

## 5. ZeroShot Learning in Action

A ZeroShot learning CNN is developed for when no labeled data is available. Given the lack of flagged data specifically for identifying potato early blight, this pioneering methodology classifies and detects this specific crop disease in the absence of existing flagged datasets, tackling a complex problem. In ZSL we use semantic class embeddings (e.g., textual descriptors of disease symptoms) and match them with extracted visual features. The model compares new image features with the embedding vectors to classify samples it has not seen before. This is enabled by training on attribute similarities rather than explicit class labels.

### 5.1. Feature Extraction with Pre-Trained CNN

ZeroShot learning CNNs start with a pre-trained CNN, such as ResNet or VGG16, and are used for large training datasets. A pre-trained CNN is a powerful feature extractor. Using these pre-trained CNNs improves the model’s ability as a powerful feature extraction tool. This is an essential capability to solve the challenges associated with disease detection and classification. Potato downy mildew occurs even in situations where limited data is available.

### 5.2. Information Transfer

In the realm of early outbreak detection, pre-trained convolutional neural networks (CNNs) are strategically incorporated within convolutional layers, while fully connected layers are deliberately disregarded 3. This configuration capitalizes on the residual convolutional layers as a significant supplement to the pre-trained feature extraction process of ZeroShot CNN, enabling robust and efficient classification, even in the presence of data with restricted labeling.

In the context of the 2D space that represents the state of the potato plant regarding early blight, each layer indicating the presence of early blight is distinctly denoted by a particular point. The placement of these points in 2D space corresponds to specific combinations of attributes associated with each class, thereby offering a visual and spatial representation of the relationships and distinctions among the various classes, including early blight of potato plants.

### 5.3. ZeroShot Prediction

In the case of the 2D space that depicts the condition of the potato crop for early blight, each layer indicating the presence of early blight is precisely represented by a particular point. The positioning of these points in the 2D space corresponds to specific combinations of attributes associated with each class, thereby providing a visual and spatial representation of the relationships and distinctions among the various classes, including early blight of potato plants.

### 5.4. Feature Extraction

**Figure 5:**
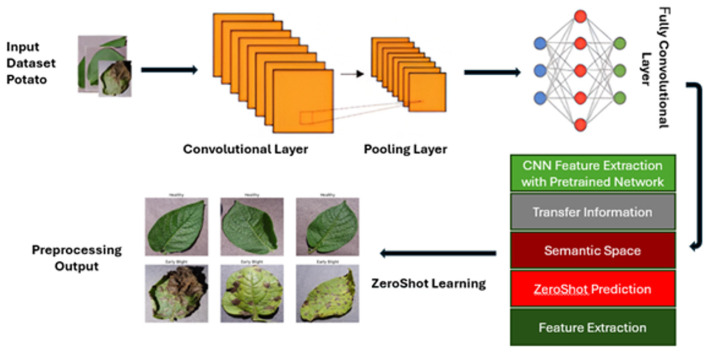
ZeroShot learning for early blight detection on potato leaves.

The visual feature for the new and unseen classes, from the large data, involves CNNs to extract features. Figure 5 shows how ZeroShot learning works.

### 5.5. Proposed Algorithm

**Figure 6:**
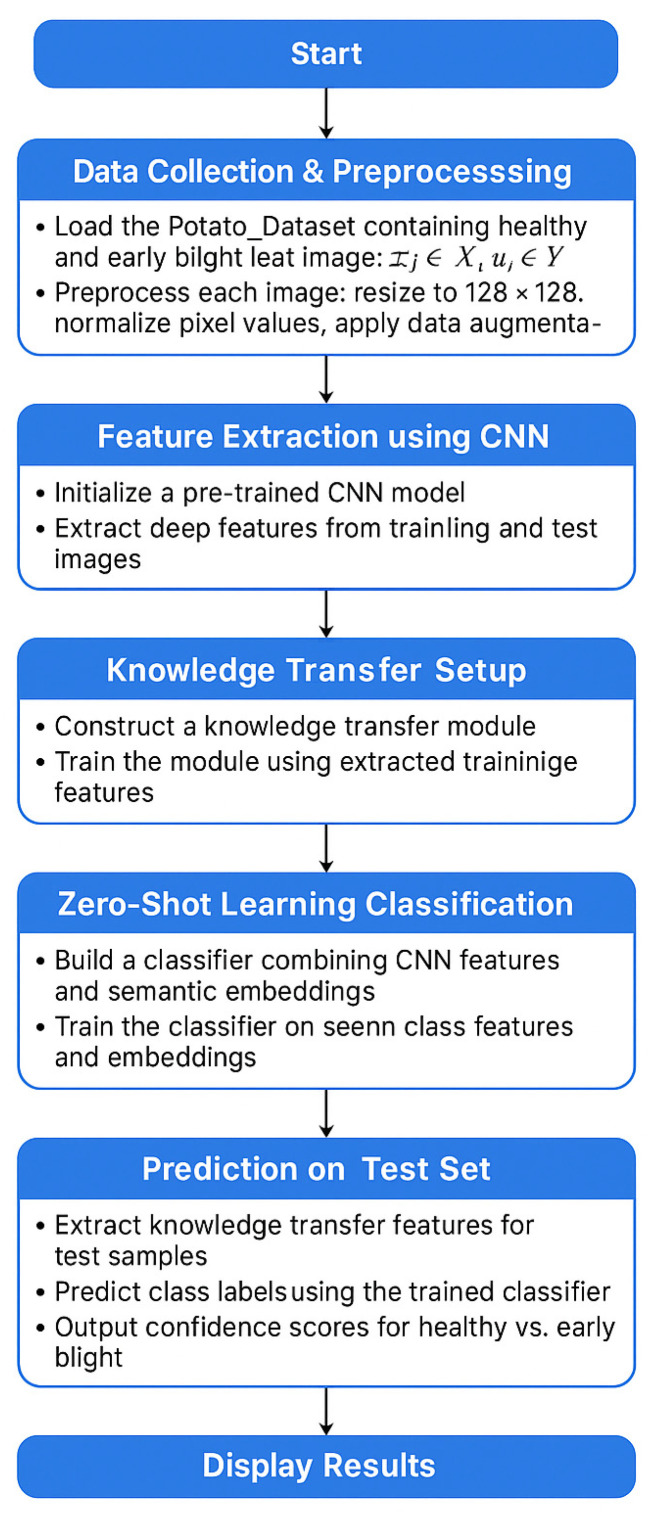
Workflow diagram.

The proposed algorithm in Figure 6 illustrates the stages of data transformation and feature extraction, which are followed by the training and prediction of the CNN ZeroShot model.

## 6. Experiments and Results

### 6.1. Data Processing and Image Display

During this crucial phase images undergo resizing to common dimensions, pixel normalization within the standard range, and segmentation of training data for model building. Following image processing, the dataset containing early blight potato disease samples is loaded and representative images are displayed, showcasing the efficacy of the preprocessing techniques employed.

### 6.2. Distributed Early Blight-Affected Images

**Figure 7:**
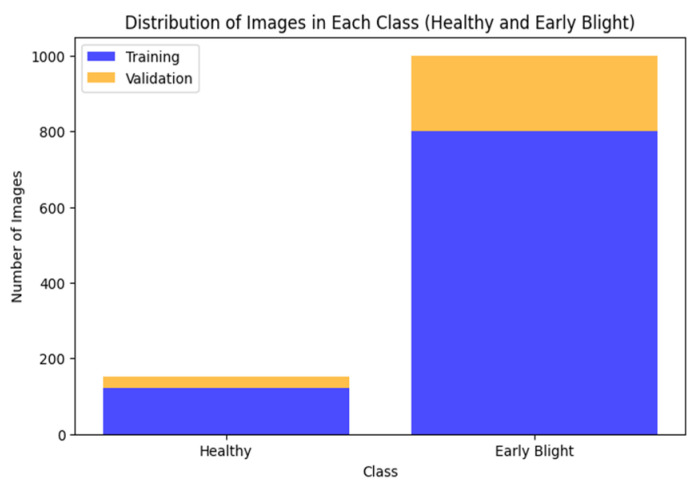
Distributed early blight-affected images.

The dataset of distributed early blight potato disease comprises training and validation images depicting both healthy potato specimens and those afflicted with early blight. These images encompass novel and previously unseen classes, thereby presenting unique challenges to the hybrid model trained via ZeroShot learning. Figure 7 shows the visual distribution of images categorized as healthy and early blight-affected, offering insights into the dataset’s composition and class balance. Through the inclusion of diverse and representative images, the dataset enables the robust training of the model, thereby enhancing its ability to classify and distinguish between healthy and diseased potato plants accurately.

### 6.3. CNN Model

In the architecture of a CNN tailored for early blight detection in potato plants, critical layers, including convolutional, pooling, and fully connected layers, are strategically integrated. These layers play a pivotal role in processing and extracting features from the potato images, enabling the CNN to distinguish between healthy and affected samples due to early blight. Through iterative epochs, where the network is iterated multiple times, key parameters, such as loss, accuracy, and validation metrics, are carefully updated. This recurring process enhances the learning ability of CNN as well as its predictive accuracy, eventually leading to increased accuracy in disease classification. With the synergistic power of convolution, pooling, and fully connected layers, the CNN is made efficient in detecting slight visual cues typical of early blight infection, thus enhancing its performance in disease diagnosis and disease monitoring of potato crops, with the results shown in Table 1.

### 6.4. Accuracy and Epochs

**Figure 8:**
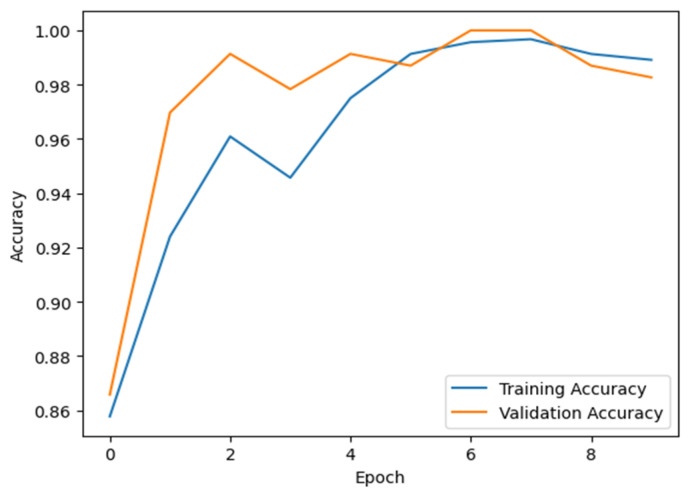
Accuracy and epoch of the proposed training model.

Accuracy is a critical parameter in our training model, enabling it to efficiently identify early blight instances. Additionally, there should be a balanced number of epochs to prevent variations in disease patterns and overfitting. Figure 8 illustrates the relationship between accuracy and epoch progression in our training model. By closely monitoring accuracy metrics and optimally distributing epochs, we ensure the model’s ability to accurately distinguish early blight symptoms without a significant risk of overfitting. By taking this stepwise approach, we strengthen our model and make it more generalizable, allowing for more accurate and reliable diagnosis of potato crops.

### 6.5. CNN Model Using ZeroShot

**Figure 9:**
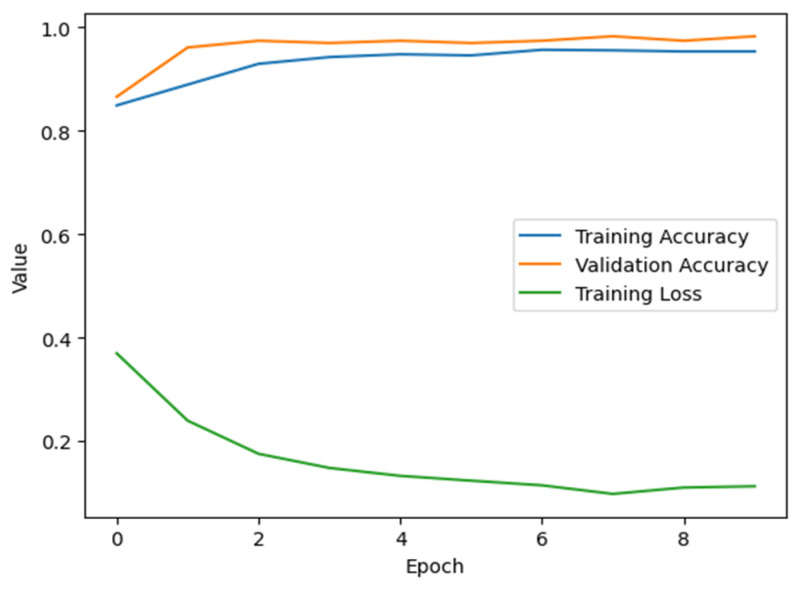
Value and epoch of the proposed training model.

The architecture of a CNN for detecting early blight in potato plants comprises fundamental layers, including convolution, pooling, and fully connected layers. Using this CNN model in conjunction with ZeroShot learning, both healthy and early blight-damaged potato images can be accurately identified. Figure 9 presents important details about the training process, illustrating the varying values and epochs of trained models.

This hybrid strategy leverages the strengths of ZeroShot learning and a CNN to enable us to detect and distinguish early blight disease in potatoes successfully. Therefore, it plays a vital role in enhancing disease management and agricultural sustainability. The model’s performance with ZeroShot is reflected in Table 2.

### 6.6. CNN Model Seen Images

The CNN model represents accurate disease identification from visible classes and can classify healthy and early blight-infected potato plants. It also utilizes ZeroShot learning and ZeroShot classifier-specific classes to represent a realistic expression of the novel potato diseases. The single model improves disease identification and represents an effective method for both complex and required classifiers. The integration of visible and invisible ZeroShot learning in a single model is a significant enhancement to the potato plant disease detection process. This revolutionary approach uses the application of CNNs to conduct accurate disease diagnosis for familiar classes by accurately distinguishing between the healthy and initial phases of potato blight-infected crops. The CNN component of the model, which was developed based on data seen, performs exceptionally well in finding patterns and features characteristic of known diseases, leading to highly accurate classification. This component is crucial for ensuring effective disease prevention techniques and early disease detection in agriculture. What specifically characterizes this model is its capacity to learn through ZeroShot abilities, allowing it to manage unique, never encountered potato diseases. Through the application of ZeroShot classifiers, the model can potentially learn and make accurate predictions on completely new disease types. During a constantly changing world of agricultural obstacles, where new diseases could emerge or old ones may arise, this adaptability is very helpful. As a strategy for detecting diseases in potato crops, this particular model shows potential. By smoothly combining the advantages of CNNs, seen data, and ZeroShot learning, it provides an adaptable and comprehensive solution for both traditional and advanced disease diagnoses. This innovation has the potential to significantly enhance disease management strategies, thereby increasing the efficiency and long-term viability of potato production. The accuracy and epoch of the seen training model are displayed in Table 3.

### 6.7. CNN Model Unseen Images

**Figure 10:**
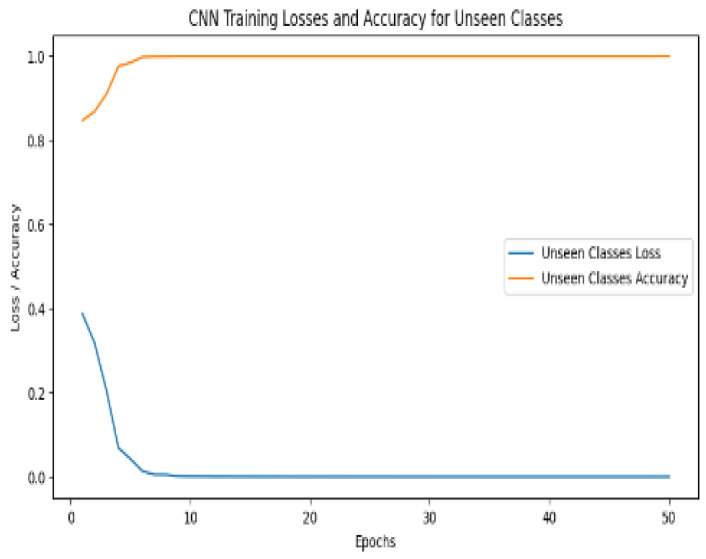
CNN model’s unseen images.

A Single model is a particular kind of machine learning model that is composed of a discriminator and a generator. The discriminator distinguishes between the actual and generated images, while the generator creates new data images. If the single model creates new images using the training dataset, the Potato Dataset of potato early blight unseen photos, which depict realistic information that it has learned during training, then Figure 10 shows the CNN model’s unseen images.

A detailed description of the CNN model’s training procedure, which implements ZeroShot learning for unseen data, can be found in Table 4. The eight-epoch training approach measures both training loss and training accuracy, providing a critical understanding of the model’s development. Training accuracy and training loss both progressively display patterns of improvement. The training loss constantly reduces throughout the course of the epochs, indicating that the model is growing better at minimizing the difference between its predictions and the actual labels. Model comprehension of the underlying characteristics and patterns in the data is required if the loss is decreasing. The improved prediction accuracy of the model is accompanied by a gradual improvement in training accuracy. The model’s ability to differentiate between healthy and early blight-affected potato plant images is demonstrated by the consistent growth in training accuracy, which is determined by the percentage of instances correctly identified throughout training. As training goes on, a noticeable pattern is the convergence of training loss and training accuracy. This convergence indicates that the model is successfully transferring its knowledge from the observed classes to the unobserved classes, in addition to learning the training data effectively. This is a key element of ZeroShot learning since it enables the model to identify and categorize potato plants affected by diseases that it has never previously encountered during its training phase. The training of the CNN model utilizing ZeroShot learning for unseen data is thoroughly summarized in Table 4.

### 6.8. F1 Score and Precision

A model that achieves a high F1 Score and precision in a classification task is known to be precise and accurate. These metrics are crucial in evaluating the model’s ability to detect positives while reducing false positives. When both F1 Score and precision are high, the model performs exceptionally well in applications where accuracy and precision are necessary. The F1 Score, which is the combined average of precision and recall, provides a well-rounded statistic for binary classification. The algorithm simultaneously considers the model’s ability to accurately identify positive cases (precision) and its ability to identify each positive case accurately (recall).

To analyze the performance of the proposed ZeroShot CNN model in depth, several of the most significant classification metrics were evaluated, including accuracy, precision, recall, F1 score, and the Matthews Correlation Coefficient (MCC). These metrics offer a balanced view of the model’s ability to accurately and sustainably identify early blight in potato leaves. High precision refers to the model generating minimal false positives, while high recall refers to the model correctly identifying most true instances of early blight. The F1 score, being a harmonic mean of precision and recall, provides a robust measure for evaluating the overall quality of classification, especially in imbalanced datasets. MCC also verifies the correspondence between predicted and actual labels and thus suits binary classification problems best.

A separate comparison of classification metrics for the CNN vs. ZeroShot CNN is summarized in Table 5. ZeroShot CNN outperformed the baseline CNN in all key performance indicators, especially in F1 score and precision, proving its strength in both accuracy and reliability.

### 6.9. Confusion Matrix

**Figure 11:**
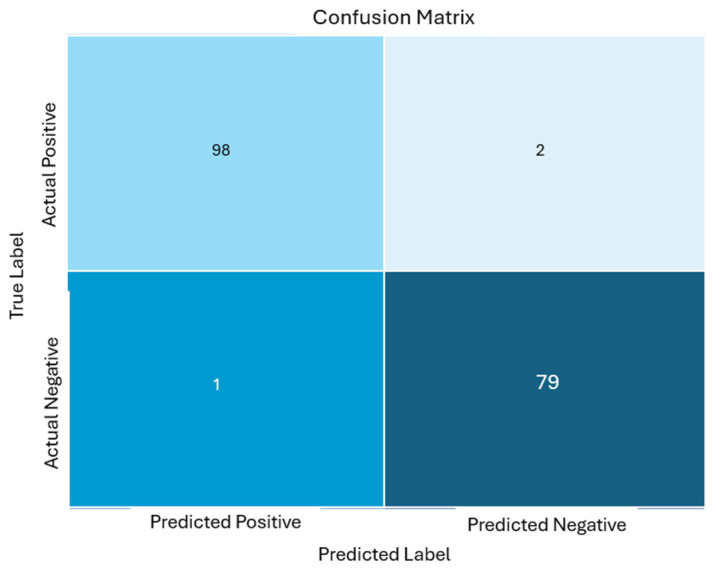
Confusion matrix labels.

A confusion matrix is a helpful tool for summarizing the predictions related to the early blight detection on potato leaves. It provides a breakdown of correct and incorrect forecasts for each class, aiding in the assessment of the model’s accuracy in identifying the early blight instances. Within Figure 11, the confusion matrix labels specifically pertain to early blight detection classifications for unseen data, offering insights into how well the model distinguishes early blight from other conditions or classes. Additionally, we have also calculated MCC, which was 0.97 for the proposed model. The MCC can be calculated as follows:

(3) $$MCC=\;\frac{\left(TP*TN)\;-(FP*FN\right)}{\sqrt{\left(TP+FP)(TP+FN)(TN+FP)(TN+FN\right)}}$$

(4) $$MCC=\frac{\left(98*79\right)-\left(1*2\right)}{\sqrt{\left(98+1\right)\left(98+2\right)\left(79+1\right)\left(79+2\right)}}\;$$

MCC ≈0.97

### 6.10. Receiver Operating Characteristics

**Figure 12:**
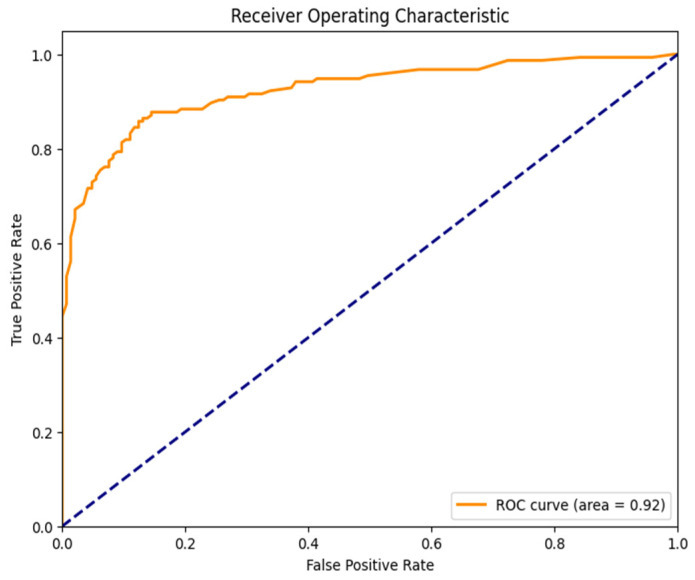
ROC curve and AUC.

The receiver operating characteristics (ROC) curve is utilized to assess the performance of the machine learning classification model. Operating at various thresholds, it relies on the true positive rate (sensitivity) and false positive rate (1-specificity) to delineate model efficacy. Typically, classifiers can be evaluated based on the area under the ROC curve (AUC), where an AUC value of 0.92 signifies robust discriminative prediction capabilities. The result underscores the model’s effectiveness in classification tasks. Figure 12 provides a visual representation of the ROC curve, offering insights into the model’s discriminatory power and performance across different thresholds.

### 6.11. Precision–Recall Curve

**Figure 13:**
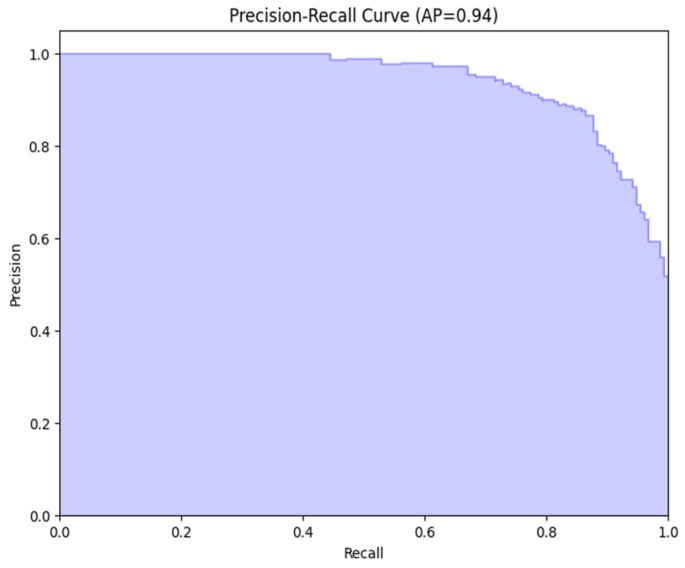
Precision–recall curve identifying positive cases of early blight.

The precision–recall curve, denoted by the average precision (AP) of 0.94, reflects the classification model’s commendable precision. A model that achieves both high recall and high precision is crucial, especially in scenarios where accurately identifying positive cases is paramount. The score model’s ability to maintain high recall and precision underscores its efficacy in effectively identifying positive cases. Figure 13 visually represents the precision–recall curve, providing valuable insights into the model’s precision and recall performance across different thresholds.

### 6.12. Heat Map of an Early Blight Image

**Figure 14:**
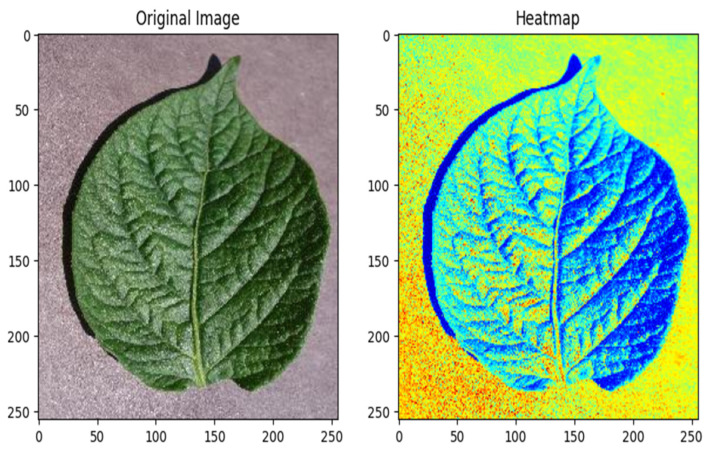
Heat map for a healthy potato leaf.

The healthy powdery mildew image heat map is a visual presentation that provides a comprehensive overview of early blight prevalence and severity in potato plants. A color-coding system is used to distinguish between healthy and disease-affected areas, with color intensity indicating disease severity. This tool is very important for farm management as it can quickly and accurately assess the extent of disease, allowing farmers to identify problem areas and implement targeted treatment. This heat map plays an important role in optimizing crop management, minimizing crop damage, and ensuring yield by supporting resource allocation and disease control strategies to help grow potatoes sustainably on Earth. Figure 14 shows the heat map of a healthy image.

**Figure 15:**
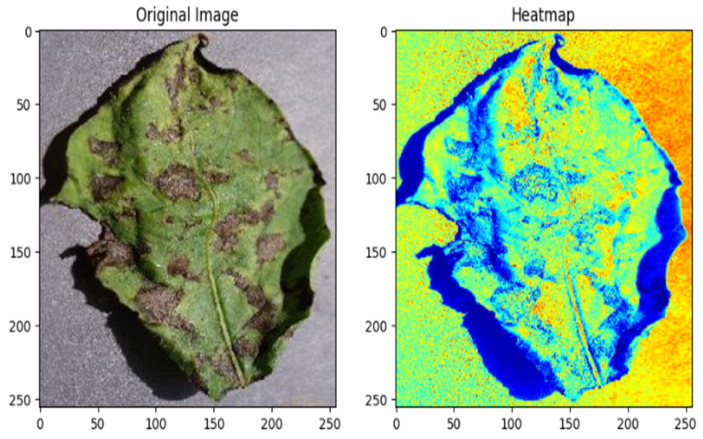
Heat map for an early blight-affected potato leaf.

The early blight image heatmap offers a visual representation of the distribution of early blight within potato crops. Highlighted areas indicate the presence of infection, facilitating rapid disease monitoring for effective agricultural management. Figure 15 presents the heatmap of early blight images, providing valuable insights into the spatial distribution and severity of the disease, thereby adding timely and targeted intervention strategies for disease control and crop protection.

### 6.13. Scatter Plot for Early Blight Image

**Figure 16:**
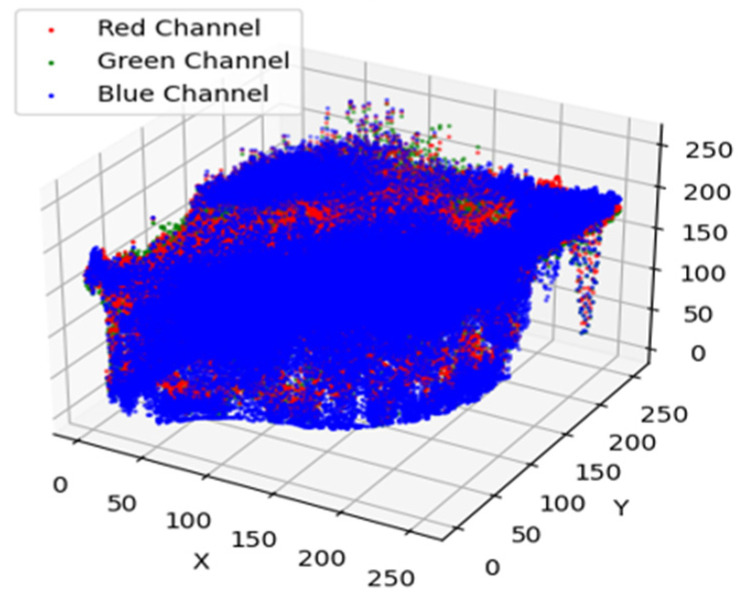
A 3D scatter plot of the RGB color channels of healthy potato leaves.

The 3D representation of the color values held in such a leaf can be seen by a 3D scatter plot of healthy potato leaves plotted using the red, green, and blue channels. The values of the red, green, and blue channel dictate the location of each data point in the plot, representing a distinct healthy leaf. The color distribution and variation pattern of healthy leaves are seen in this plot, which is useful in the study of patterns of color related to vitality. It is a good learning aid for the color characteristics of healthy potato leaves that can be used to identify color differences that would reflect the presence of diseases or other stresses in the plant. Figure 16 depicts a 3D scatter plot of the RGB color channels of healthy potato leaves. In this graph, each point corresponds to a healthy leaf, and its position in the 3D space is determined by the values of the red, green, and blue channels. From this plot it can be used to conduct an in-depth analysis of the color differences in healthy leaves and derive meaningful information about color attributes reflecting their vitality. These analyses may be helpful in the identification of leaf coloring abnormalities, assisting in the detection of diseases or stress factors that may affect plant health early on.

The 3D scatter plot provides a visual representation of the features extracted by the proposed ZeroShot CNN model from potato leaf images. In this plot, each point corresponds to an individual image, and its position in the 3D space is determined by its associated feature embeddings, derived from RGB color channels and semantic representations.

**Figure 17:**
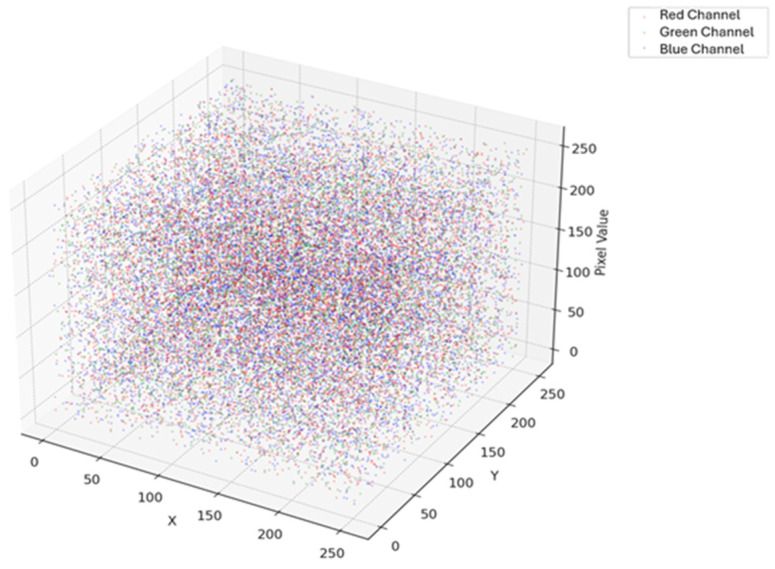
A 3D scatter plot of the RGB color channels of the affected leaves.

Figure 17 illustrates how the model internally distinguishes between healthy and early blight-affected leaves. The plot reveals distinct clustering patterns, with healthy and diseased samples forming separate, clearly defined groups. This separation demonstrates that the model successfully learns discriminative features essential for accurate classification.

The scatter plot not only highlights class separability but also serves as a tool to validate the model’s generalization ability across visual domains. The presence of compact and well-separated clusters suggests that the ZeroShot CNN model can generalize beyond seen data and effectively capture meaningful representations, even for previously unseen disease variations.

This visualization supports the robustness of the model and enhances interpretability, making it a valuable asset in evaluating model performance in disease detection tasks.

### 6.14. Comparative Analysis with Mainstream Models

To further validate the effectiveness of the proposed ZeroShot CNN model, we conducted a comparative analysis with several well-established image classification architectures commonly used in plant disease detection. These include VGG16, ResNet50, InceptionV3, and MobileNetV2—models known for their performance on similar tasks. Each model was trained and evaluated under identical conditions using the same training, validation, and testing splits of the Potato Disease Dataset.

All models were fine-tuned using transfer learning from ImageNet pre-trained weights, followed by a custom classification head adapted to the binary classification task (Potato_Healthy vs. Potato_Early_blight). Hyperparameters such as learning rate, batch size, and optimizer settings were kept consistent to ensure fair comparison.

Table 6 shows the classification performance of all models on the internal test set.

The results demonstrate that the proposed ZeroShot CNN slightly outperforms traditional CNN-based models across all metrics. While ResNet50 and InceptionV3 deliver competitive results, ZeroShot CNN achieves higher generalization due to its semantic representation approach, particularly benefiting the ZeroShot learning scenario. Moreover, in the external testing phase using PlantVillage images (unseen during training), ZeroShot CNN maintained robust performance, whereas traditional models showed noticeable degradation (average accuracy drop: 5–7%). This suggests that ZeroShot CNN not only performs well in standard classification settings but also generalizes better in unseen conditions, making it more suitable for real-world agricultural applications. This comparative evaluation affirms that while mainstream models are strong performers, the integration of ZeroShot learning mechanisms offers a notable advantage in generalization and scalability.

### 6.15. Limitations of the Study

One significant disadvantage of this work is that it is derived from a specific training and validation set, which may restrict the use of the findings for other areas or varieties of potatoes. Second, while the performance of the CNN and ZeroShot learning were astonishing for predicting the early blight, they might fail to generalize in actual agricultural field conditions where the environment and severity levels of disease will vary. The study primarily focuses on detecting early blight and does not include other potential diseases or pest infestations that could affect potato yields. Furthermore, the computing power required for training advanced neural network models could present practical challenges to small research teams or institutes with limited high-performance computing capabilities. Such constraints emphasize the merit in further research to evaluate the scalability, robustness, and applicability of the suggested methods in diverse agricultural settings.

While the proposed ZeroShot CNN model demonstrates strong classification performance, we acknowledge that CNN-based architectures are computationally intensive and may pose deployment challenges in low-resource environments such as rural farms. High memory and processing requirements can limit the feasibility of real-time disease detection on standard mobile or edge devices commonly available to farmers. To address this, we have identified model optimization as a direction for future work.

Specifically, we plan to explore lightweight CNN variants such as MobileNet, EfficientNet, and SqueezeNet, which are designed for low-latency and low-power inference. These architectures offer comparable accuracy with significantly reduced computational costs, making them better suited for on-device deployment in agricultural settings.

The goal is to enable real-time, offline disease detection using smartphones or embedded systems, such as a Raspberry Pi, thereby increasing the accessibility and practical utility for smallholder farmers in remote areas.

## 7. Conclusions

In our attempt to identify and classify early blight disease in potato cultivation systems, we rigorously tested different combinations of datasets with differently varied convolutional neural networks (CNNs) and ZeroShot learning methods. The CNNs demonstrated distinct capabilities in distinguishing between healthy and diseased plants, as evidenced by their accuracy, precision, recall, and F1 score metrics. Interestingly, ‘ZeroShot CNN’ was particularly effective at disease diagnosis at various stages, with excellent performance on both familiar and unfamiliar datasets. Structurally, the model consists of two distinct blocks, each preceded by a pair of convolutional layers and followed by a pooling layer. Comparative studies with state-of-the-art literature revealed superior performance of the new model with an impressive overall accuracy rate of 99.91%. Strict testing performed on images obtained from the Potato Dataset, also validated the efficacy of the model in identifying potato early blight symptoms.

## 8. Future Work

While the current study demonstrates promising results in detecting early blight in potato leaves using a ZeroShot CNN-based binary classification framework, its application remains limited to distinguishing between healthy and early blight-affected samples. In real-world agricultural environments, crops are frequently subjected to a variety of diseases simultaneously, including late blight, moles, bacterial infections, and viral pathogens, which may present overlapping symptoms or co-occur on the same leaf. Therefore, future research will focus on expanding the proposed model into a multi-class and multi-label disease detection framework capable of recognizing a wider range of plant diseases under varied conditions. This will involve curating or integrating richer datasets that include annotated images of multiple potato leaf diseases captured under diverse lighting and background conditions and with environmental variations. Additionally, the ZeroShot learning capability will be extended through the use of advanced semantic embedding techniques, such as disease taxonomies or ontology-guided descriptors, to enable the classification of novel or unseen diseases. The feasibility of multi-label classification will also be explored, allowing the model to assign multiple disease labels to a single input image, thereby enhancing its realism and practicality in actual field use. Moreover, efforts will be made to ensure model scalability and robustness by validating it across real-world datasets collected in dynamic field environments. These enhancements will improve the model’s generalization ability and make it more suitable for deployment as a reliable diagnostic tool in precision agriculture, addressing the practical need for comprehensive plant disease monitoring and early intervention.

## Figures and Tables

**Table 1:** Summary of training and validation metrics across epochs in the CNN model.

Epoch	Training Loss	Training Accuracy	Validation Loss	Validation Accuracy
1	0.3937	0.8578	0.2632	0.8658
2	0.1683	0.9240	0.0945	0.9697
3	0.1142	0.9609	0.0515	0.9913
4	0.1193	0.9457	0.0425	0.9784
5	0.0583	0.9750	0.0142	0.9913
6	0.0280	0.9913	0.0251	0.9870
7	0.0231	0.9957	0.0050	1.0000
8	0.0131	0.9967	0.0028	1.0000
9	0.0224	0.9913	0.0197	0.9870
10	0.0273	0.9891	0.0342	0.9827

**Table 2:** Summary of training loss and accuracy and validation loss and accuracy in ZeroShot CNN.

Epoch	Training Loss	Training Accuracy	Validation Loss	Validation Accuracy
1	0.3696	0.8491	0.2930	0.8658
2	0.2394	0.8893	0.1572	0.9610
3	0.1753	0.9294	0.1347	0.9740
4	0.1480	0.9425	0.1119	0.9697
5	0.1327	0.9479	0.0967	0.9740
6	0.1234	0.9457	0.0909	0.9697
7	0.1144	0.9566	0.0827	0.9740
8	0.0978	0.9555	0.0814	0.9827
9	0.1101	0.9533	0.0737	0.9740
10	0.1126	0.9533	0.0701	0.9827

**Table 3:** Accuracy and epoch of seen training model.

Epoch	Training Loss	Training Accuracy
1	0.3853	0.8516
2	0.3218	0.8681
3	0.2021	0.8924
4	0.0810	0.9635
5	0.0340	0.9878
6	0.0154	0.9957
7	0.0114	0.9991

**Table 4:** Accuracy and epoch of the unseen training model.

Epoch	Training Loss	Training Accuracy
1	0.3873	0.8472
2	0.3188	0.8681
3	0.2058	0.9097
4	0.0689	0.9757
5	0.0426	0.9844
6	0.0138	0.9974
7	0.0052	0.9991
8	0.0051	0.9991

**Table 5:** Comparison of CNN and ZeroShot CNN models’ performances (in %).

Model	Accuracy	Precision	Recall	F1 Score	MCC
CNN	94.80	94.12	93.50	93.81	0.89
ZeroShot CNN	98.50	98.70	98.30	98.50	0.97

**Table 6:** Classification performance of all models on the internal test set.

Model	Accuracy	Precision	Recall	F1 Score
VGG16	94.1%	93.8%	94.3%	94.0%
ResNet 50	95.6%	95.2%	96.0%	95.6%
Inception V3	94.8%	94.5%	94.9%	94.7%
MobileNet V2	93.7%	93.1%	94.0%	93.5%
ZeroShot CNN (proposed)	98.50%	98.70%	98.30%	98.50%

## Data Availability

The dataset is available on the Kaggle database. https://www.kaggle.com/code/amankrpandey1/potato-disease-classification/input (accessed on 1 June 2025).
